# Make America Healthy Again: a medico-legal and public health analysis of a politicized health initiative

**DOI:** 10.3389/frhs.2025.1632180

**Published:** 2025-07-01

**Authors:** Camilla Cecannecchia

**Affiliations:** Section of Legal Medicine, Department of Clinical and Experimental Medicine, University of Foggia, Foggia, Italy

**Keywords:** health policy, health equity, legal medicine, bioethics, public health

## Introduction

In early 2025, the U.S. Department of Health and Human Services unveiled the MAHA initiative under the leadership of Secretary Robert F. Kennedy Jr. Promoted as a comprehensive response to America's chronic disease epidemic, the initiative targets environmental toxins, aims to reform dietary policies, and emphasizes holding corporations accountable for health-related outcomes (1). While MAHA addresses genuine issues—including the rising prevalence of obesity, diabetes, and autoimmune disorders—it presents a model that some view as ideologically driven and not fully grounded in scientific consensus (2, 3) This analysis seeks to explore both the initiative's strengths and its potential shortcomings, particularly concerning its medico-legal and ethical implications.

Furthermore, while MAHA highlights the need to confront the increasing prevalence of chronic diseases, there are concerns about the scalability and feasibility of its proposed solutions. For instance, the initiative's focus on environmental toxins, while important, may be difficult to implement at a national level due to the complexity of regulating pollutants across diverse industries and sectors. Additionally, addressing chronic disease prevention through environmental and dietary changes requires widespread public education and awareness, which may take years to fully realize. A more comprehensive approach would involve collaboration between local, state, and federal governments, alongside private sector engagement, to foster a more integrated and sustainable model for public health improvement (4, 5).

## Public health rhetoric and MAHA's causal framework

MAHA posits that increases in chronic conditions—particularly in children—are predominantly due to exposure to environmental toxins, processed foods, and regulatory neglect. Although environmental and dietary factors undeniably contribute to non-communicable diseases (NCDs), attributing causality primarily to external toxins overlooks the multifactorial nature of chronic illness. Scientific evidence indicates that conditions such as obesity, type 2 diabetes, and attention-deficit/hyperactivity disorder (ADHD) arise from complex interactions between genetic predisposition, lifestyle behaviors, socioeconomic factors, and environmental exposures (6–8). Framing chronic disease etiology in a largely deterministic and externalized manner may inadvertently reduce the perceived importance of behavioral and preventive interventions. This oversimplification can obscure more holistic public health strategies and divert attention from evidence-based, multifaceted approaches.

It is also important to consider that while environmental toxins and processed foods play a role in the development of chronic conditions, the emphasis on these factors alone may not adequately address the root causes of disparities in health outcomes across different demographic groups. For example, people from low-income or marginalized communities often face a combination of socioeconomic stressors—such as inadequate healthcare access, limited educational opportunities, and living in food deserts—that compound the impact of poor environmental conditions. Thus, any public health initiative, including MAHA, must account for these complex social determinants of health to prevent deepening existing health inequities. A broader and more inclusive approach to policy development could foster better long-term outcomes (9, 10).

## Legal and institutional implications

MAHA's narrative often includes critiques of federal health institutions such as the Centers for Disease Control and Prevention (CDC) and the Food and Drug Administration (FDA), portraying them as entities compromised by corporate interests. While transparency and accountability are essential in regulatory science, persistent delegitimization of public health authorities may erode public trust and reduce adherence to guidelines and vaccination programs (11, 12). MAHA's focus on corporate accountability, while aimed at addressing valid concerns regarding industry influence on public health policy, may inadvertently lead to an increase in litigation based on alleged harms that lack substantiated causal evidence. Legal actions might be initiated against food, pharmaceutical, or chemical companies on the basis of personal anecdotes, unverified claims, or misinformation, rather than conclusions drawn from methodologically rigorous scientific research. For example, assertions linking childhood vaccinations to autism or associating genetically modified foods with autoimmune diseases—despite being unsupported by the prevailing epidemiological consensus—could gain legal traction if evidentiary standards for causation are weakened. The adjudication of such cases without strict scientific criteria risks establishing legal precedents that diverge from established scientific understanding, potentially burdening judicial systems and discouraging innovation across the health, pharmaceutical, and food sectors (13). Additional concerns have been raised regarding the qualifications of individuals reportedly appointed to federal advisory roles under the MAHA framework. The report appears to include appointees with backgrounds in environmental activism, legal advocacy, or alternative medicine, many of whom may lack formal training in medicine, public health, or epidemiology. The inclusion of such individuals in influential policymaking roles could undermine the scientific integrity and methodological soundness of public health decision-making, particularly in areas requiring specialized expertise in disease prevention, risk assessment, and regulatory science (14). On the other hand, the legal implications of MAHA's corporate accountability framework raise the question of whether such measures could inadvertently lead to a chilling effect on innovation. If pharmaceutical and food companies are subject to increased litigation without robust evidence of direct harm, companies might redirect resources away from research and development toward legal defense, thereby slowing down advancements in treatments and preventive technologies. This potential shift in focus could undermine the very goals of public health policy by diverting attention and resources from proactive disease prevention and public education to legal battles. Therefore, a careful balance must be struck between holding corporations accountable and ensuring that the focus remains on evidence-based solutions to health issues (15, 16).

## Politicization of scientific uncertainty

A notable feature of MAHA is its use of contested or refuted scientific claims to justify policy interventions. For instance, theories suggesting links between vaccines and autism—despite being thoroughly discredited by the scientific community—are occasionally echoed within MAHA-affiliated rhetoric (16, 17). This exploitation of scientific uncertainty can foster public confusion, alienate substantial sections of the population, and impede timely responses to emerging health threats. Furthermore, amplifying unproven hypotheses risks diverting research funding from validated priorities and may contribute to regulatory stagnation in areas such as vaccine policy or food safety reform (17).

Another consequence of politicizing scientific uncertainty is the potential undermining of public trust in the scientific process itself. By emphasizing controversial or disproven theories, there is a risk of eroding the credibility of scientific consensus, particularly in health-related fields where public trust is crucial for the success of prevention programs and vaccination campaigns. The politicization of science can further alienate the public, making it more difficult to achieve consensus on other health issues, such as climate change or the regulation of harmful substances. Consequently, the rhetoric surrounding MAHA needs to be more carefully crafted to avoid divisiveness and promote a more collaborative approach to public health policymaking based on rigorously collected and evaluated evidence (18, 19).

## Equity and accessibility in health interventions

Several MAHA proposals, such as exclusive use of organic foods in public school meals or bans on specific additives, aim to improve health outcomes but may also inadvertently exacerbate disparities. For families and institutions with limited resources, these measures could lead to increased costs and reduced food security, disproportionately affecting lower-income populations (20). Health interventions must account for economic feasibility and cultural accessibility. Policies rooted in well-intentioned but economically unrealistic standards may lead to unintended nutritional deficits, especially among children who rely on school-provided meals. In addition, public skepticism toward conventional medical practices, when not grounded in critical scientific appraisal, can result in reduced uptake of preventive care such as vaccines or cancer screenings, further widening health inequities (21).

Moreover, while initiatives like MAHA aim to improve public health outcomes, their focus on high-cost solutions such as organic food could inadvertently exacerbate health disparities, especially in rural and underserved urban areas where access to such foods is limited. The proposed policy changes need to be more nuanced to ensure they are economically viable for a broader segment of the population. There is also the potential for cultural resistance, as dietary preferences and traditions vary greatly across different groups in the United States. If public health policies are to be truly effective, they must be adaptable to different cultural contexts and incorporate input from communities affected by such policies to ensure they are both inclusive and equitable in their execution (22, 23).

## Ethical dimensions of parental choice

MAHA strongly advocates for parental autonomy in medical decision-making, especially regarding childhood vaccinations and dietary practices. While autonomy is a cornerstone of bioethics, it must be balanced with the principle of beneficence and the need to protect vulnerable populations. Informed refusal of preventive interventions—when based on misinformation—can undermine herd immunity and endanger public health. Legal tensions may arise when such refusals intersect with child welfare standards, potentially leading to claims of medical neglect or custodial intervention (23). Ethical public health policy requires navigating this delicate balance between respecting personal liberties and safeguarding collective well-being.

Additionally, while the principle of parental autonomy is crucial, it is essential to examine the ethical tension between individual rights and public health responsibilities. In cases where misinformation is prevalent, such as the vaccine-autism controversy, the impact of parental decisions extends beyond individual families to affect the health of the broader community. Public health experts argue that widespread misinformation can compromise population-level immunity (i.e., the level of immunological protection within a community that reduces the risk of disease transmission, including to those who cannot be vaccinated), putting vulnerable populations at greater risk. This raises important ethical questions about the limits of parental autonomy in the context of collective well-being, and whether certain health interventions should be mandated in the face of public health emergencies to safeguard society at large (24–27).

## Conclusion

The “Make America Healthy Again” initiative reflects a growing public desire to confront the root causes of chronic disease and corporate influence in health policy. While MAHA presents a compelling narrative that challenges established norms, its approach often relies on oversimplification, contested scientific claims, and emotionally charged rhetoric. A sustainable and equitable health reform agenda should be firmly anchored in evidence-based practice, transparent governance, and respect for both scientific integrity and social equity. To that end, policies must be guided by interdisciplinary collaboration, rigorous research, and a nuanced understanding of complex health determinants—avoiding the pitfalls of politicization, reductionism, and regulatory overreach. To effectively address the complexities of chronic disease prevention and healthcare reform, the MAHA initiative will need to incorporate a more interdisciplinary approach, drawing not only from environmental and dietary science but also from social, economic, and behavioral sciences. Collaboration between policymakers, healthcare professionals, industry experts, and the public will be critical to ensure that the initiative's goals are met without overlooking the complexities of human health. Moreover, it is notable that the MAHA initiative devotes limited attention to well-established determinants of chronic disease such as tobacco use and alcohol consumption, factors supported by a robust body of epidemiological evidence and a precedent of effective regulatory intervention. This omission raises significant concerns regarding the overall comprehensiveness of the initiative and the coherence of its public health priorities.

As the initiative progresses, ongoing evaluation and flexibility will be necessary to adapt to emerging evidence and evolving public health needs. Only by engaging with these broader dimensions can MAHA avoid becoming a one-size-fits-all solution and instead serve as a model for comprehensive and sustainable health reform.
